# Depression detection using virtual avatar communication and eye tracking

**DOI:** 10.16910/jemr.16.2.6

**Published:** 2023-08-06

**Authors:** Ayumi Takemoto, Inese Aispuriete, Laima Niedra, Lana Franceska Dreimane

**Affiliations:** University of Latvia, Riga, Latvia; Riga Stradins University, Riga, Latvia; Tohoku University, Sendai, Japan

**Keywords:** Eye tracking, Saccades, Virtual avatar communication, Depression detection, Human-computer interaction

## Abstract

Globally, depression is one of the most common mental health issues. Therefore, finding an effective way to detect mental health problems is an important subject for study in human-machine interactions. In order to examine the potential in using a virtual avatar communication and eye tracking system to identify people as being with or without depression symptoms, this study has devised three research aims; 1) to understand the effect of different types of interviewers on eye gaze patterns, 2) to clarify the effect of neutral conversation topics on eye gaze, and 3) to compare eye gaze patterns between people with or without depression. Twenty-seven participants - fifteen in the control group and twelve in the depression symptoms group - were involved in this study and they were asked to talk to both a virtual avatar and human interviewers. Gaze patterns were recorded by an eye tracking device during both types of interaction. The experiment results indicated significant differences in eye movements between the control group and depression symptoms group. Moreover, larger gaze distribution was observed when people with depression symptoms were discussing neutral conversation topics rather than those without depression.

## Introduction

During the first two decades of the 21st Century, mental, or mood disorders (12) have been two of the most common health problems negatively affecting the quality of life as well as the longevity of the global population; these problems include depression, schizophrenia, anxiety disorder, and bipolar disorder (27). Recent studies have identified major depressive disorders as amongst the leading causes of disability worldwide (27) with more than ten categories of sub-types (47). Since the COVID-19 pandemic began in 2020, the number of identified cases of patients suffering from depression has been increasing day by day (9; 50). These swiftly increasing patient numbers have resulted in the challenge of the majority of national healthcare systems being overwhelmed by COVID-19 patients and limitations caused by the pandemic (2). In particular, the healthcare sector has been affected by these stressful situations and the increase in workloads during the pandemic (16). Thus, new forms of healthcare services have been developed since the pandemic began (2). Telemedicine, or telehealth systems which support booking consultations, seeing doctors remotely or actual diagnosing have been developed more actively to avoid the risk of contracting a nosocomial, or secondary infection (51).

Scientific studies since the 1970s and through to the 2020s have reported that non-verbal behavioral signals, such as eye contact, head angle, and mouth angle, can reflect depression, anxiety, or negative emotions in human behavior (7; 15; 17; 32; 34; 65). Waxer (65) reported that there was no difference in the amount of eye contact frequency between people with, or without depression symptoms; however, people with depression made eye contact with others for about 25% longer than people without depression symptoms. These results showed that people with depression gazed at their conversational partners for a shorter period of time than people without depression. Cummins et al. (7) summarized behavioral markers of depression, including non-verbal and verbal features, such as speaking rate, pitch, eye movements, social interactions, and facial activities. Their study showed that depressed people generally demonstrated a lack of energy dynamics such as a decrease in eye movements, social interactions, vocal pitch, and facial expressions, and there was an increase in slower speaking rate, visual fixation, and pause rates. Eye movement was reported to be one of the criteria that detected depression and anxious, or negative emotions (24; 36; 63). For instance, it was suggested that fewer saccades and longer fixation duration in patients with depression were observed, compared to participants without depression, when they were observing black and white pictures which included nature, social situations, still objects, and meaningless images (e.g. arrayed lines, blurred noise) (36). Li et al. (16) concluded that these features of people with depression were consistent with capturing less information than people without depression, in that the relevant brain area in the frontal lobe processed information more slowly than people without depression. Wang et al. (63) reported that in patients with major depression, bipolar depression, and bipolar mania, eye movement patterns were similar as well as smaller saccade amplitude, compared to people in the healthy-control group while they were observing pictures which included human faces, natural landscape, man-made environments, and computer-generated images. The results interpreted by Wang et al. (63), highlight that people with depression had some dysfunction under free-viewing, fixation stability and smooth pursuit tasks and showed an acquisition and processing of information different from that of people without depression.

However, when exploring the different ways facial expressions and eye-gaze have been employed as methods for studying mental health and depression, questions about the effects of different types of interviewers or the actual conversation topics, on gaze, facial expression, emotions, or other non-verbal information of depressed people remain unanswered.

To investigate the effect of virtual avatar communication on people with depression, this research focused on three aims: 1) to understand the interviewers’ effect on eye movements; 2) to clarify the effect of conversation topics on eye movements; 3) to investigate the criteria which reflect depression.

Past studies have investigated the interviewers’ effects on impression and rapport with participants. Gratch et al. (19) summarized the effect of types of interviewers on behavior and initial impression and reported that people use twice as many filled pauses (e.g. expressions such as ”uh”, ”um”, and ”well”) while talking to the virtual avatar as opposed to when they are talking to a live interviewer. Furthermore, other past studies reported that a virtual agent, whose ethnicity is similar to that of the interviewee, has proven to have a great impact in changing their action (45); however, in self-reported research, gender and ethnicity appearance did not have much impact (48). In addition, Gratch et al. (20) reported the type of interviewers affected emotional bonds: a virtual avatar that reacts using positive feedback induces stronger rapport than a human with positive feedback. Thus, the first research aim of this study is to understand the interviewers’ effects on eye movements in both people with and without depression symptoms during interaction with human and virtual avatar interviewers.

It was reported that clinical interviews were primarily used for studies focusing on detecting moods, or mental disorders, such as depression (4; 22), post-traumatic stress disorder (PTSD) (3), or attention deficit hyperactivity disorder (ADHD) (23; 54). Guohou et al. (22) reported that the performance of a depression detection model was better in problem-related questions, such as depression, personality, and emotion. On the other hand, another past study suggested that clinical interviews, including disclosure of feelings and problem-solving, induced more anxiety, depression, and behavioral fear than unrelated conversation topics (5). The second aim is to clarify the effect of conversation topics on eye movements in both people with and without depression symptoms.

It has been reported in several studies that people with depression symptoms tend to make fewer social interactions. The lack of eye contact, for example, is induced in the depression symptoms group (13; 29; 53). Zhang et al. (67) reported that people with depression indicated eye movement anomalies; in particular, reduced saccade amplitude, shorter scan path length, and lower saccade velocity in the free-viewing test were observed. Furthermore, Crawford et al. (6) reported that the saccade frequency in people with depression symptoms is less than those without depression in visual stimuli tasks; on the other hand, fixation duration in people with depression symptoms is longer than those without depression in visual stimuli tasks (59). The final aim of this study is to investigate the criteria for gaze patterns that reflect depression while talking to the virtual avatar about non-clinical interview topics.

In this study, twenty-seven participants - fifteen in the control group and twelve in the depression symptoms group - were asked to talk to each human, or virtual avatar interviewer on each negative, or neutral, conversation topic through a monitor; meanwhile eye movements were recorded.

## Methods

The experiments were conducted with participants interacting with a 3-dimensional cartoon-type virtual avatar and a recorded human interviewer through a monitor. Participants performed conversation tasks with each interviewer. In this section, participants’ traits, interviewers, and experimental protocols are introduced, and then the details of the implemented analysis are reported. This research focused on the analysis of gaze patterns between different types of; 1) participants (people with or without depression), 2) interviewers (human or virtual avatar interviewer), and 3) conversation topics (neutral or negative topics). Thus, this paper presents the results of the eye gaze pattern.

### Participants

All the participants were native Latvian speakers. For determination of the small sample size, an a priori power analysis (G*Power ver 3.1 (14)) indicated that the required sample size was a mere twelve people for the control group (the score of PHQ-9 is lower than 10) and depression symptoms group (the score of PHQ-9 is 10 or higher). Participants for both the control group (N = 17) and the depression symptoms group (N = 13) were recruited and screened using PHQ-9 through a Social Networking Service (SNS). All participants provided written, informed consent before the experiment and received a gift worth approximately 12 USD. All participants answered PHQ-9 on the day that they participated in the experiment, and a male participant in the depression symptoms group, whose PHQ-9 answered through SNS was higher than the cut-off score was found to have it lower. In addition, a female and a male participant in the control group, who experienced a technical issue in the middle of experiments, were excluded from all of the analysis. These participants’ population is the same as our previous research paper (60).

### Surveys

In this study, three surveys were used to measure participants’ characteristics and the effect of each experimental condition: 1) Positive and Negative Affect Schedule measured the effect of types of interviewers and conversation topics; 2) Patient Health Questionnaire-9 measured the level of depression and was used to classify participants into two groups, the control and depression symptoms groups; 3) International Personality Item Pool-Five Factor Model-50 was used to identify the characteristics of participants. These are the details of each survey.

1. Positive and Negative Affect Schedule (PANAS)

Positive and Negative Affect Schedule (PANAS) are widely used throughout psychology studies to measure mood induction (21; 64) and consist of twenty-item scales to measure both positive and negative affects. Each item can be scored from 1 (not at all) to 5 (very much). The reliability of this survey to measure the emotional effect was reported in many different types of medical situations, and the psychometric properties of the scale were clarified in clinical individuals with anxiety, depressive, and adjustment disorders (10).

2. Patient Health Questionnaire-9 (PHQ-9)

A Patient Health Questionnaire-9 (PHQ-9) is commonly used to screen for depression, and scores can range from 0 to 27, as each of the nine items can be scored from 0 (not at all) to 3 (nearly every day). The PHQ-9 has demonstrated reliability and validity and is highly adaptable to patients with MDD in psychiatric hospitals. It is reported as a simple, rapid, effective, and reliable tool for screening and assessing the severity of depression (57). Kroenke et al. (33) reported that a PHQ-9 score ≥ 10 had a sensitivity of 88 % and also a specificity of 88 % for major depression. Furthermore, Manea et al. (38) reported that there are no significant differences in sensitivity and specificity for cut-off scores between 8 and 11. For the purposes of this study a score of 10, which is the most common, was used as a cut-off score. Participants answered PHQ-9 in Latvian (44) before starting the experiment.

3. International Personality Item Pool – Five Factor Model – 50 (IPIP-Big5)

International Personality Item Pool – Five Factor Model – 50 (IPIP-Big5) (18; 56) is widely used throughout Psychology studies to classify and compare personality traits in many types of languages (66; 68). IPIP-Big5 correlates with the Big-Five Inventory (28) scale and the reliability of the five factors has been reported to be high (68). Furthermore, past studies reported that IPIP-Big5 was studied in people with depression (30). The IPIP-Big5 translated and verified by Pērkona and Koļesovs (46) based on Perepjolkina and Reņģe (43) and Schmitt et al. (52) was used in this experiment. The questionnaire consists of a fifty-item scale, and each item can be scored from 1 (Disagree strongly) to 5 (Agree strongly). The five basic dimensions of personality were based on the study published by Strus et al. (56).

### Apparatus

The interviewers were presented on a monitor (Lenovo, 2880 × 1620 pixels, 34.31 × 19.30 cm) at a viewing distance of 60cm and controlled by a native Latvian member of the experiment team through a Unity game engine in the same room. Eye movements of participants were monitored using Tobii Pro Nano with a 60Hz refresh rate calibrated before each session by the Tobii python SDK.

### Experimental setup

**Figure-01:**
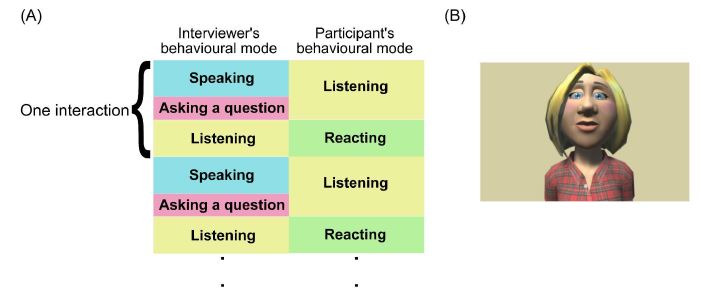
(A) Experimental flow and behavior modes of participants and interviewers and (B) the appearance of the virtual avatar interviewer (The figure was created by Toon people ver 3.1 which is a Unity asset produced by JBGarrazaUnity (62)/CC BY 4.0)

Interaction of the conversation task involves roughly structured dialogues between the participant and the interviewer. Each session has two modes based on participants’ behavior – the listening mode where the interviewer led the conversation with a closed-ended question based on the topic, and the reacting mode where the participant was asked to answer the question for about five seconds (Figure-01-(A)). Two members of the experiment team were in the same room as the participants and controlled the system based on participants’ reactions and offered a break between sessions. In the case of the human interviewer, if participants talked for more than ten seconds, the video which was playing was automatically stopped until the system was moved to the next interaction. Participants performed four sessions (two sessions were neutral topics and other two sessions were negative topics) so that each session had thirty interactions where an interaction consisted of listening and reacting mode in the participant’s behavioral mode (Figure-01-(A)). The order of the combination of the conversation topics and the interviewers’ types were assigned randomly to participants. Before starting the main session, participants had practiced talking to the virtual avatar interviewer about animals in five interactions. In order to clarify the effects of interviewers and conversation types, motivated by the method of past study by Gratch et al. (19), participants were asked to fill in PANAS before and after each session.

### Interviewers

Two types of interviewer were prepared; an animated type of virtual avatar and a human to emphasize the differences between them. For the determination of a virtual avatar’s appearance, many research journals already reported that there is no significant difference between a human-like and an animated avatar, age, gender, and ethnicity of virtual avatars in frustration levels, preference, and the level of rapport (25; 48; 45). Several different types of virtual avatars’ pictures were developed that resembled the general Latvian appearance – light skin tone, blue eyes, and blonde hair, current casual clothing, and hairstyle, then students in the Department of Psychology were asked to rate their impression of them using the 5-point Likert scale (e.g. 1. Friendly – 5. Unfriendly) based on the previous paper by Pratt et al., (45), and the virtual avatar which had the highest score for that impression was chosen. The virtual avatar utilized is Toon People ver 3.1 which is a Unity asset produced by JBGarraza (62). Figure-01-(B) shows the virtual avatar interviewer’s appearance. The voice data were produced by NCH Software (41) and Hugo.lv which was used as the online text-to-speech application (35) to convert the written text into spoken words. The virtual avatar interviewer was computed to blink four or three times per ten seconds based on the average natural human blinking ratio (40; 61) and to move the mouth based on sentences. In the listening mode, interviewers were talking to participants and asking a question, and participants were listening to this. In the reacting mode, interviewers were nodding, and participants were answering the questions. In the case of the human interviewer, the videos were prepared in that a native Latvian had spoken the same sentence as the virtual avatar and afterward the human interviewer nodded for approximately ten seconds which was twice as long as the length the participants were previously asked to talk. These, then, were played in order, and participants interacted with the video through a monitor.

### Conversation task

Two types of conversation topics were prepared in Latvian - negative topics (war and loneliness) as it has been reported these topics have a high impact on vocal, visual, and verbal features to detect depression (22), and neutral topics (gardening and traveling). Each topic consisted of thirty sentences with closed-end questions (the answer can be ’yes’ or ’no’) which asked about their experience (e.g. Have you ever been to Latvia?, Have you ever seen firefighting?), knowledge (e.g. Do you know places to see cherry blossoms?, Do you know the Israel-Palestina conflict?), and themselves (e.g. Do you like traveling?, Did you feel lonely in the COVID-19 pandemic?). Conversation scripts and questions were reviewed by psychology and cognitive psychology researchers to ensure that participants felt fewer negative emotions (anxiety, depression, and behavioral fear).

### Eye tracking analysis

From the recorded eye positions of the participants’ right and left eyes, three types of data were obtained: saccades’ frequency, fixation duration, and gaze distribution. All data were analyzed using MATLAB (MathWorks, Natick, MA) and Python. Fixation points were detected by using the EyeMMV toolbox (31); the system used a two-step spatial dispersion threshold for fixation identification. First, the average horizontal and vertical coordinates were computed as the length between the average point and the record was greater than the first allowed value (we set two degrees in this study), and, if the distance was greater than two degrees, a new fixation cluster was generated; Second, the distance between the mean point and each record in each cluster was calculated, so that, if the distance for a record was greater than one degree, that record was not used as the fixation. The minimal fixation duration was 100 msec in this study.

The number of saccades in each session was used as a metric; specifically, the number of saccades occurring in a second was computed and used as the value of the saccades’ frequency. The average duration of each gaze fixation point in each session was computed and the values were used as the fixation duration. Furthermore, the average distance between the center of the display and each gaze fixation point was computed in each session and the values were used as the gaze distribution. The distance between the center of the display and the interviewer’s eyes was approximately 4 cm. In the human interviewer, eye tracking data, before stopping the videos, were used for analysis because the effect of stopping the video had to be considered.

### Statistical analysis

In the analysis of PANAS and reaction duration, a mixed design three-way analysis of variance (ANOVA) within/between interaction was conducted with the types of participants, interviewers, and conversation topics as the main factors. In the analysis of eye gaze patterns, four-way ANOVA within-between interaction was conducted with types of participants, interviewers, conversation topics, and behavioral modes as the main factors. In the ANOVAs of this study, a Huynh-Feldt correction was applied when the assumption of sphericity was not met by the Mendoza test. A 95% confidential interval (CI) was computed based on Loftus and Masson’s procedure, and a p-value of 0.05, which is the most common as a cut-off (8; 26), was used as ’statistically significant’.

## Results

A post-hoc analysis was conducted by G*Power (14) to confirm sufficient statistical power (Power = .945). The characteristics of participants in each group was indicated in Table 1. This section reports the results of eye movements between different types of participants, interviewers, conversation topics, and behavioral modes (listening, or reacting mode in participants’ behavioral mode) to investigate which criteria were affected by the types of participants, and whether there were any effects caused by the different types of conversation topics or interviewers.

**Table 1.** Participants’ characteristics

	Control group	Depression Symptoms group
N	15	12
Female number	8	9
Age range	20 – 48 (30.75 ± 8.90)	20 – 47 (28.25 ± 8.69)
PHQ-9 score range	2 – 9 (5.47 ± 1.92)	10 – 23 (14.08 ± 4.83)

**Figure-02:**
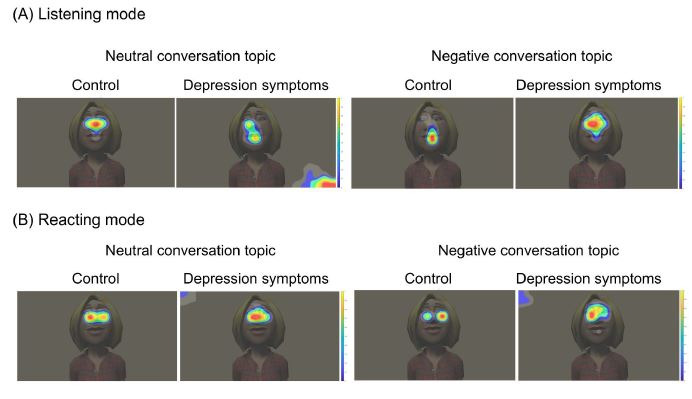
Exemplary heat maps for comparison between the control and depression symptoms group in (A) listening mode and (B) reacting mode.

As the heat map figures (Figure-02) indicate, there are different eye gaze patterns between the control and depression symptoms groups. The collected data highlighted that people in the depression symptoms group tended to look away from the area of the interviewer’s face. Eye movements such as saccades’ frequency (the number of saccades occurring in a sec), fixation duration (the duration of each gaze fixation point), and gaze distribution (the distance between the center of the display and each gaze fixation point), were analyzed in order to understand the effect of experimental conditions on types of participants.

### Saccades’ frequency

**Figure-03:**
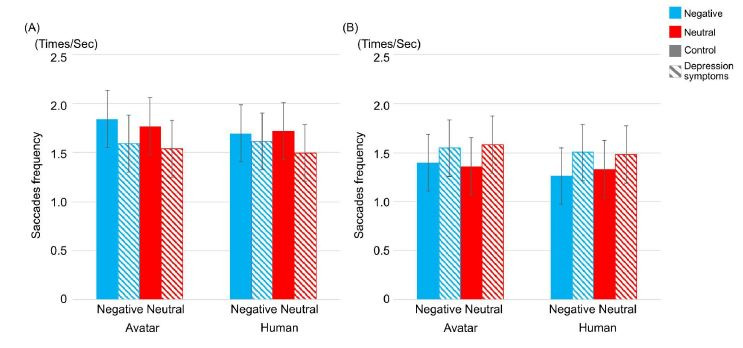
Average of saccades’ frequency (Times/sec) between types of participants, types of interviewers, and types of conversation topics in reacting mode (A) and listening mode (B). Striped pattern boxes indicate the data of the depressed symptoms group, and solid boxes are the control group. Error bars indicate 95% CI.

Figure-03 indicates the result of saccades’ frequency in each experimental condition and each type of participants. Interaction between types of participants and behavioral modes showed a certain trend toward significance (*F*(1,25) = 3.445, *p* = .075, η^2^ = .031), and there was also a significant interaction between types of behavioral modes and conversation topics (*F*(1,25) = 5.149, *p* = .032, η^2^ = .001). Simple effects for types of behavioral modes and conversation topics were computed in order to understand the effect of types of conversation topics. In both types of behavioral modes, there was no significant difference between negative and neutral conversation topics. On the other hand, to clarify whether saccades’ frequency is one of the criteria required to detect depression, simple effects for types of participants x types of behavioral modes were computed. As a result, there was a different tendency between the control group and the depression symptoms group: there was a significant difference between reacting and listening mode in the control group (F(1,14) = 6.8608, p = .0202, η^2^ = .1346), and saccades’ frequency was larger in reacting mode than in listening mode (reacting mode, M = 1.76 (time/sec), SD = 0.51 (time/sec); listening mode, M = 1.34 (time/sec), SD = 0.56 (time/sec)); however, in the depression symptoms group, no significant difference was observed. Thus, the analysis supported the claim that it is possible to effectively use saccades’ frequency as one of the criteria to detect depression.

### Fixation duration

**Figure-04:**
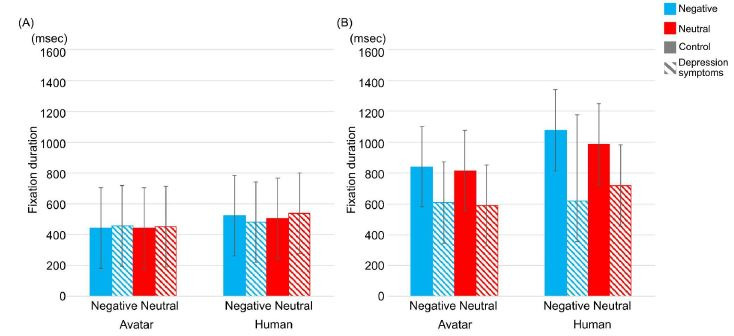
Average of fixation duration (msec) between types of participants, interviewers, and conversation topics in reacting mode (A) and listening mode (B). Striped pattern boxes indicate the data of the depressed symptoms group, and solid boxes are the control group. Error bars indicate 95% CI.

There were no significant interactions between types of participants, interviewers, conversation topics, and behavioral modes; however, there was significance in the main effects of types of behavioral modes and interviewers (*F*(1,25) = 8.697, *p* = .007, η^2^ = .088; *F*(1,25) = 5.355, *p* = .029, η^2^ = .010, respectively). In types of behavioral modes, the fixation duration was longer in the listening mode than in the reacting mode. Furthermore, in types of interviewers, fixation duration was longer in the human interviewer than in the avatar interviewer (Figure-04). With regard to results, it is not possible for the fixation duration to be a criterion in classifying the control group or the depression symptoms group.

### Gaze distribution

**Figure-05:**
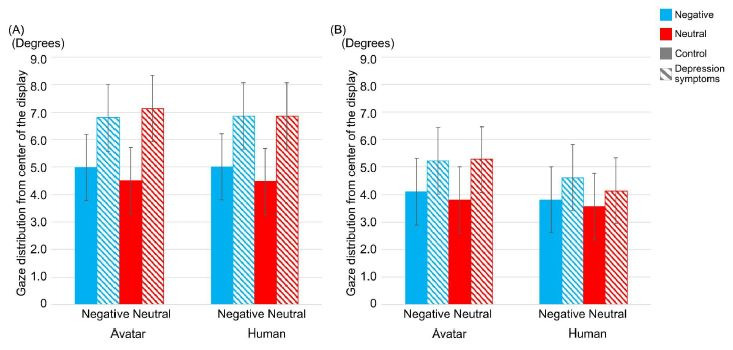
Average of gaze distribution (degrees) on the display between types of participants, interviewers, and conversation topics in the reacting mode (A) and the listening mode (B). Striped pattern boxes indicate the data of the depressed symptoms group, and solid boxes are the control group. Error bars indicate 95% CI.

Figure-05 shows the result of gaze distribution from the center of the display (degrees). First, the result which related to the differences in types of interviewers was reported as the interaction between types of behavioral modes and interviewers (*F*(1,25) = 7.865, *p* = .001, η^2^ = .003). In the simple effect for types of behavioral modes and interviewers interaction, there was significance in the gaze distribution between reaction- and listening mode in both types of interviewers (virtual avatar: *F*(1,25) = 23.463, *p* < 0.001, η^2^ = .056, human: *F*(1,25) = 34.085, *p* < 0.001, η^2^ = .155). However, there was no significant difference between types of interviewers in both types of behavioral modes, suggesting that gaze distribution is affected by types of behavioral modes, but is not affected by types of interviewers. Next, the interaction between types of participants, behavioral modes, and conversation topics and between types of participants and behavioral modes were significant (*F*(1,25) = 9.370, *p* = .005, η^2^ = .001, *F*(1,25) = 4.828, *p* = .038, η^2^ = .014, respectively), and the main effects of types of participants and behavioral modes were also significant (*F*(1,25) = 4.660, *p* = .041, η^2^ = .103, *F*(1,25) = 32.179, *p* < 0.001, η^2^ = .094, respectively). In simple effects between types of participants and behavioral modes, the result indicated that the gaze distribution in the depression symptoms group was larger than in the control group in reacting mode (control group, M = 4.74 (degrees), SD = 1.51 (degrees); depression symptoms group, M = 6.91 (degrees), SD = 3.24 (degrees); *F*(1,25) = 5.762, *p* = .024, η^2^ = .166); therefore, gaze distribution can be a criterion used to effectively detect depression. Moreover, the results were analyzed separately in both negative and neutral conversation topics for greater understanding. Three-way ANOVA between types of participants, interviewers, and behavioral modes as the main factors were conducted in each type of conversation topics. First, in the negative conversation topics, there were no significant interactions; thus, gaze distribution while contributing to the negative conversation topics did not reflect depression. On the other hand, in neutral conversation topics, the interaction between types of participants and behavioral modes was significant (*F*(1,25) = 7.093, *p* = .013, η^2^ = .021, statistically significant as p < 0.025 after Bonferroni correction). In simple effects for types of participants x types of behavioral modes in neutral conversation topics, there was significance between the control group and the depression symptoms group during the reacting mode (*F*(1,25) = 6.923, *p* = .014, η^2^ = .197, statistically significant as p < 0.025 after Bonferroni correction). Specifically, in the reacting mode, gaze distribution in the depression symptoms group was approximately twice as large as in the control group in neutral conversation topics (control group: M = 4.49 (deg.), SD = 1.38 (deg.), the group with depression symptoms: M = 6.98 (deg.), SD = 3.50 (deg.)). However, there is no significant difference between the types of participants in the listening mode.

## Discussion

The effect of types of interviewers and conversation topics on eye movements in both the control and depression symptoms groups was examined. In this section, the results are interpreted with respect to the three aims presented in the Introduction as follows; 1) to understand the effect of different types of interviewers on eye gaze patterns, 2) to clarify the effect of conversation topics on eye gaze patterns, and 3) to compare eye gaze patterns between people with or without depression while talking to the virtual avatar about non-clinical interview topic.

### Human and virtual avatar interviewers’ effect on eye gaze patterns

The comparison of eye movements between types of interviewers such as a human, or virtual avatar, revealed that types of interviewers did not affect eye gaze patterns in both the control and the depression symptoms groups. 

There was a statistically significant difference in the types of interviewers in the fixation duration; namely, the fixation duration for the human interviewer was longer than for the virtual avatar interviewer in both types of participants. There appeared to be no reports to compare the human and virtual avatar interviewers’ effect on fixation duration; however, Manor and Gordon reported that the fixation duration, while participants were looking at the human face, was longer than when they were looking at abstract geometric figures (39). It is possible that participants would recognize the virtual avatar face as figures; thus, the fixation duration for the virtual avatar interviewer was shorter than the human interviewer. However, the limitation of this study is that an animated virtual avatar as a virtual avatar interviewer and a recorded video as a human interviewer was used, thus, the effect of a human-like virtual avatar and real-human interviewers is still unclear.

### Negative and neutral conversation topics’ effects on eye gaze patterns

The effect of types of conversation topics on eye movements revealed differences in gaze distribution. In neutral conversation topics, gaze distribution in the depression symptoms group was larger than the control group when they were answering the questions from the interviewers. It would appear that few research papers have reported the effect of conversation topics on eye movements. On the other hand, articles (including pictures and text) on different topics have different effects on people with, or without depression. People without depression tend to focus on positive pictures and text more than the negative; however, people with depression tend to look at the negative article rather than the positive one because the interest of the negative article in people with depression was higher than the positive article (49). Furthermore, Suslow et al. (58) highlighted a summary of research papers that looked at eye tracking research in people with, or without depression. They concluded that people with depression dedicated more attention to sad faces and dysphoric pictures than people without depression, and people with depression looked less at happy faces than people without depression. Based on these previous studies, it is a possible theory that the depression symptoms group would be less interested in neutral conversation topics rather than negative conversation topics, and it would encourage their eyes to wander more on the display. The limitation of this study is that a closed-end question (participants can answer “yes” or “no”) was used to control the experimental duration, thus it is unclear whether an open-ended question (participants cannot answer with “yes” or “no”) has any effect in people with depression.

### Comparison of eye movements between control and depression symptoms groups

The main aim of this research is to clarify which eye movements’ parameters are the criteria required to effectively detect depression; saccades frequency, fixation duration, or gaze distribution. Statistical values were computed for each parameter. The conclusion is that gaze distribution can be a criterion required to effectively detect depression. It is important to explain the interpretation in saccades’ frequency and gaze distribution.

First, with regard to the saccade frequency, the saccades’ frequency during the reacting mode is larger than during the listening mode in the control group; however, there is no significant difference in the depression symptoms. It appears that there are no studies that compared saccades’ frequency between reacting and listening modes. One previous study reported that eye movements were more focused on the objects during convergent thinking tasks than divergent thinking tasks (37); namely, saccades’ frequency increased in the divergent thinking task rather than in the convergent thinking tasks. In these results, in the listening mode, participants would focus on listening to, and understanding, the interviewers’ talk; thus, it was supposed that they would not be induced to think in multiple ways. In the reacting mode, however, participants were asked to answer the questions based on their experience, or opinions. It was believed that the reacting mode would work the same as divergent thinking tasks because participants would think about, and reply to, the questions from different aspects. The different effects between reacting and listening modes would encourage the difference in saccades’ frequency; namely, saccades’ frequency in the reacting mode was larger than in the listening mode.

Next, gaze distribution in the depression symptoms group is basically larger than in the control groups; the differences between control and depression symptoms groups during the reacting mode in neutral conversation topics are especially noteworthy. Bailly et al. (1) reported that people look at interviewers’ faces when they are talking. They, especially, tend to stare at the eyes when they are talking but they tend to look at the mouth when they are listening. Interviewers’ eyes were set approximately four degrees away from the center of the display in this experiment, and the average of gaze distribution in the control group was less than five degrees. This shows that they mostly focused on interviewers’ faces, and, thus the results are consistent with Bailly et al. (1). On the other hand, the gaze distribution in the depression symptoms group was larger than in the control groups in both reacting and listening modes. There appear to be no studies comparing gaze distribution between a control and depression symptoms group. However, several studies have summarized social skills in control and depression symptoms groups. These have suggested that the frequency of eye contact with the interviewer in the depression symptoms group is less than in the control group (29; 53). This research regarding gaze distribution in the depression symptoms group shows results that are generally greater than in the control group; namely, they would not look at interviewers’ eyes in either reacting or listening duration. This is consistent with past studies’ results. Taking into account the gathered evidence, this study interpreted that looking into the interviewers’ eyes, or faces would be uncomfortable for the depression symptoms group.

Three other limitations were considered in this study. First, classification experiments using machine learning or AI were not conducted in this study. Several scientific papers have reported the classification of people with or without depression using machine learning algorithms including random forests, logistic regression, and support vector machines based on physiological data such as gaze patterns, data observed in EEG, and galvanic skin response in visual tasks, and the classification accuracies were greater than 70 % (11; 55). In further studies, classification experiments using computer science methodologies would be required in order to detect depression symptoms in virtual avatar communication with neutral conversation topics.

Secondly, gaze data were collected by a research screen-based eye tracker, Webcam-based eye tracking was not attempted in this study. However, since eye tracking with webcams generally provides 2 to 5 degrees of accuracy, the same trend in gaze distribution may be observed with eye tracking using webcams.

Finally, in this study, the first threshold was set at two degrees as the spatial dispersion threshold for fixation identification, although these values could affect the higher level of gaze analyses. However, one of the previous studies on the EyeMMV algorithm reported that applying different spatial thresholds (0.7 to 1.3 degrees) integrated into the algorithm did not show significant differences (42).

In conclusion, this study has indicated that there was no emotional effect from the different types of interviewers, but there was a significant difference between people, with or without depression in eye gaze patterns, especially, saccades’ frequency and gaze distribution. Furthermore, in the gaze distribution, neutral conversation topics induced more significance between people with and without depression than negative conversation topics. Considering this and our previous studies (60), types of interviewers has no effect on the emotions of people with depression, and people with depression have different patterns of non-verbal behavior in neutral conversation topics.

### Ethics and Conflict of Interest

The author(s) declare that the contents of the article are in agreement with the ethics described in http://biblio.unibe.ch/portale/elibrary/BOP/jemr/ethics.html and that there is no conflict of interest regarding the publication of this paper. This study was approved by the Ethics Committee of the University of Latvia in accordance with the Declaration of Helsinki (approval number: 30-47/18).

### Acknowledgements

This research was supported by European Regional Development Fund (ERDF) for Post-doc projects grant agreement No 1.1.1.2/VIAA/4/20/668. We would like to thank you Ms.Anna Digna Dubrovska for translating all of documents from English to Latvian and Dr. Aleksandrs Kolesovs and Ms.Kitija Perkona for sharing Big5 in Latvian. The pre-registration of this research has been registered in Open Science Framework (OSF) (Registration DOI: 10.17605/OSF.IO/B9DNE).
